# Outcome measures based on digital health technology sensor data: data- and patient-centric approaches

**DOI:** 10.1038/s41746-020-0305-8

**Published:** 2020-07-23

**Authors:** Kirsten I. Taylor, Hannah Staunton, Florian Lipsmeier, David Nobbs, Michael Lindemann

**Affiliations:** 1 Pharma Research and Early Development, Roche Innovation Center Basel, F. Hoffmann-La Roche Ltd, Grenzacherstrasse 124, 4070 Basel, Switzerland; 2 Faculty of Psychology, University of Basel, Missionsstrasse 60/62, 4055 Basel, Switzerland; 3 Patient-Centered Outcomes Research, Biometrics, Product Development, Roche Products Limited, Hexagon Place, 6 Falcon Way, Shire Park, Welwyn Garden City, AL7 1TW UK

**Keywords:** Outcomes research, Parkinson's disease, Diagnostic markers, Drug development, Neurological manifestations

## Abstract

Digital health technology tools (DHTT) are technologies such as apps, smartphones, and wearables that remotely acquire health-related information from individuals. They have the potential advantages of objectivity and sensitivity of measurement, richness of high-frequency sensor data, and opportunity for passive collection of health-related data. Thus, DHTTs promise to provide patient phenotyping at an order of granularity several times greater than is possible with traditional clinical research tools. While the conceptual development of novel DHTTs is keeping pace with technological and analytical advancements, an as yet unaddressed gap is how to develop robust and meaningful outcome measures based on sensor data. Here, we describe two roadmaps which were developed to generate outcome measures based on DHTT data: one using a data-centric approach and the second a patient-centric approach. The data-centric approach to develop digital outcome measures summarizes those sensor features maximally sensitive to the concept of interest, exemplified with the quantification of disease progression. The patient-centric approach summarizes those sensor features that are optimally relevant to patients’ functioning in everyday life. Both roadmaps are exemplified for use in tracking disease progression in observational and clinical interventional studies, and with a DHTT designed to evaluate motor symptom severity and symptom experience in Parkinson’s disease. Use cases other than disease progression (e.g., case-finding) are considered summarily. DHTT research requires methods to summarize sensor data into meaningful outcome measures. It is hoped that the concepts outlined here will encourage a scientific discourse and eventual consensus on the creation of novel digital outcome measures for both basic clinical research and clinical drug development.

## Introduction

Digital health technology tools (DHTTs) are digital tools that remotely and frequently acquire health and disease-related data from individuals (see Table 1 for a list of terms and definitions used here). DHTTs are becoming a standard part of clinical research studies in which patients collect two basic modalities of sensor data^1–7^: (1) sensor data can be captured while patients perform specific tasks designed to measure key symptoms (“active tests”) and (2) sensors may monitor human behavior passively (“passive monitoring”). Active tests can be administered, e.g., using tablets, smartphones, and to a limited extent also smartwatches. Passive data can be collected using a wide range of devices such as smartphones, smartwatches, wearables, beacons, nearables, and smart patches. Digital sensors can sample behavior at a frequency many times higher than is possible during traditional in-clinic testing. Thus, there are many advantages to the use of this technology in clinical research. Firstly, information is collected from patients much more frequently than would be possible at infrequent clinic visits, with the potential to increase the signal to noise and increasing reliability and validity^8^ and the measures’ ability to detect change over time^9^. Secondly, the acquired data are objective: while trained healthcare professionals have a wealth of knowledge with which to characterize and treat patients’ symptoms, the reliance on such ratings necessarily adds a subjective component to assessments including possible increased interrater variability and decreased reliability (e.g., see ref. ^10^). Thirdly, data are collected in the environment in which individuals actually live and work, generating ecologically valid measurements, i.e., ones which approximate the real world. A fourth key advantage is the amount of sensor data generated (e.g., via continuous 50 Hz sampling), promising the phenotypic characterization of patients with an unprecedented breadth and precision. These advantages have led to DHTTs being used with increasing frequency in clinical trials and observational studies to complement traditional clinical outcome assessments (COA) and biomarkers. However, one of the chief open questions now facing the field is what to do with this vast amount of sensor data, i.e., how can patients’ sensor data be integrated into one or several outcome measures which meaningfully recapitulate the clinical status of the patient^5,11^?

**Table 1:** Terms and definitions.

Term	Definition
Digital health technology tools (DHTTs)	Technologies such as apps, smartphones, and wearables that remotely acquire health-related information from individuals
Digital outcome measure	An outcome measure derived from data captured with a digital health technology tool
Data-centric digital outcome measure	A digital outcome measure that uses a data-driven approach to summarize those sensor features maximally sensitive to the concept of interest
Patient-centric digital outcome measure	A digital outcome measure that uses patient insights to summarize those sensor features that are optimally relevant to patients’ functioning in everyday life
Disease progression digital outcome measure	A digital outcome measure that quantifies change in a health condition over time
Case-finding digital outcome measure	A digital outcome measure used in a general practitioner setting to aid in the detection of individuals with a potential disease
Active test	A standardized task during the execution of which sensor data are collected with digital health technology tool
Passive monitoring	The recording of human behavior or physiology with a digital health technology tool which does not require an individual’s active participation
Ecologically valid measurement	A measurement recorded by a digital health technology tool during the participant’s everyday life or in conditions that approximate the participant’s everyday life
Sensor feature	A characteristic of sensor data derived by an algorithm

The optimal method to summarize DHTT data will depend on the intended use of the digital outcome measure. Here, we focus on the possibility of aggregating symptom data, as opposed to a single sensor feature reflecting an individual sign or symptom (e.g., see ref. ^12^). We focus on aggregated data because digital outcome measures incorporating the measurement of multiple symptoms may better capture global disease burden in syndromes such as Parkinson’s disease (PD) with heterogeneous profiles of symptoms, each of which may progress at different rates at different disease stages. A holistic assessment of symptoms may allow for the detection of changes in symptoms in a heterogeneous population both across individuals, and over time within individuals.

Below, we present two roadmaps to develop outcome measures based on DHTT data. Both focus on measures of disease progression for use in longitudinal clinical research studies. Applications in case-finding and treatment monitoring are considered summarily. The first “data-centric” approach is designed to maximize sensitivity to detect disease progression. The second, “patient-centric” approach is designed to measure progression of symptoms which patients consider most meaningful to their daily lives. To exemplify the roadmaps, we reference a DHTT tool designed to measure core motor symptoms in individuals with PD^13^.

## A framework to develop digital outcome measures

One of the main challenges faced by researchers collecting DHTT data is how to combine the abundance of sensor data in a meaningful way to produce a score that is tailored to both the symptoms under investigation (e.g., core motor symptoms of PD) and the context in which the score will be used (e.g., monitoring disease progression and evaluating treatment response)^14,15^. For DHTTs, this process would entail a data preparation phase, resulting in sensor features (e.g., stride length), followed by a feature selection phase, resulting in a set of sensor features tailored to the intended use, and finally a model build and validation stage producing the final digital outcome measure^12^. While a digital outcome measure using the recommended procedure has yet to be published, it is currently envisaged that the resultant digital outcome measure will provide a single score which integrates a variety of sensor feature data from the DHTT.

**Fig. 1:**
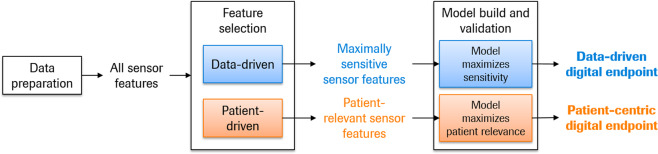
Procedure to develop digital outcome measures. Signal processing generates sensor features which may be selected in a data-driven or patient-centered procedure to generate maximally sensitive or patient-relevant digital health technology outcome measures, respectively.

Two fundamentally different approaches can be used to generate digital outcome measures: one driven by the data, and one driven by the “patient’s voice”, i.e., those sensor features which best reflect what patients consider to be important for their daily functioning. The ensuing outcome measure could rely on either one of these approaches in isolation, or a combination of the two^16^. Data-centric approaches summarize those sensor features maximally sensitive to the concept of interest, e.g., case-control differences for case-finding, or disease progression for disease monitoring, and are commonly used in experimental academic research. Another goal exists in clinical drug development: to measure how a patient feels, functions or survives in evaluating responses to an intervention. In this latter case, the DHTT sensor data must be relevant to patients’ functioning in everyday life^11^. This patient-centric approach is recommended by the health authorities (e.g., ref. ^17^) for the determination of whether the drug under investigation has meaningfully improved patients’ daily lives. The sensitivity of the outcome measure to its intended purpose can be further increased during the model building procedure (see Fig. 1).

Below, we present roadmaps for the development of data-centric and patient-centric digital outcome measures. Throughout, we exemplify these roadmaps with a technology developed to remotely and frequently monitor motor symptoms in PD, i.e., the Roche PD Mobile Application v2 (refs ^13,18^, see also refs ^19,20^). We note that this approach can be applied to any DHTT and corresponding disease area, e.g., actigraphy data to quantify sleep quality^21^ or photoplethysmography data from, e.g., the Apple Watch to quantify cardiovascular health^22^, as described in the Conclusions section. Briefly, the Roche PD Mobile Application v2 provides patients with a dedicated smartphone and requests them to use it to perform brief, daily “active tests” of motor functioning, and to place the smartphone in a provided running belt and wear it as they go about their daily lives (“passive monitoring”). Active testing and passive monitoring may detect quantitatively and qualitatively different levels of symptomatology, i.e., maximal capacity on the one hand (i.e., active testing), and day-to-day functioning on the other (passive monitoring)^23,24^. Throughout both the active tests and passive monitoring, the smartphone sensors (i.e., tri-axial accelerometer, gyroscope, magnetometer) sample patients’ movement patterns at circa 50 Hz, while a GPS receiver samples dereferenced location coordinates once per second, touch screen interactions at 60 Hz and sound at 44.1 kHz (“raw data”). The resultant data are then segmented, quality controlled (QC’ed) and analyzed to generate sensor features (e.g., turn speed) which are ultimately combined to generate a single score representing clinical status. Since both roadmaps assume that data segmentation, QC, and sensor feature extraction are complete, these processes are described first.

### Data preparation

Raw sensor data must first be segmented into meaningful sets of information. This would entail, e.g., extracting that segment of sensor feature data corresponding to the time period when a patient was performing a rest tremor test task with a smartphone, or the periods of time in which the patient was going about their daily lives, excluding, for example, device recharging. Segmented raw sensor data then undergo a QC process to ensure that the data reflect the intended activity (e.g., smartphone was not resting on the table during a balance test or during putative passive monitoring segments). This process relies on bespoke algorithms that capitalize on known sensor signals associated with the intended versus unintended activities. Segmented raw sensor data not surviving QC are not further analyzed. Passive monitoring data should then undergo a second segmentation step, in which the streams of raw sensor data may be analyzed to segment periods of time according to the putative activity that took place during the corresponding period (e.g., climbing stairs, walking), for example, via machine learning models developed based on independent and publicly available passive monitoring data^25^. This process enables the quantification of intuitive activity types, e.g., sit-to-stand transitions or the time spent walking^13^. Features are then extracted from the segmented and QC’ed sensor data, each of which represent an aspect of the symptom under investigation. For example, in both active gait tests and passively monitored gait, potential sensor features are step frequency variability to measure gait quality and turn speed to estimate bradykinesia and rigidity.

Prior to modeling, the robustness of individual sensor features must be examined. In particular, the variability of sensor feature values as a function of the sensor type and model should be quantified to determine the number of measurements required to achieve an acceptable level of reliability. Also test–retest studies should be conducted to quantify different biological sources of variability, in particular disease-independent (e.g., difference between sensor feature data acquired from two consecutive time periods) and disease-dependent (e.g., difference between sensor feature data acquired on different days) sources of variability. Appropriately aggregated sensor features (e.g., all sensor feature values over 2 weeks^13^) represent the building blocks for the DHTT outcome measure, which we recommend to further screen for measurement noise as part of the score-building process described below.

### A roadmap to develop a data-centric digital outcome measure

**Fig. 2:**
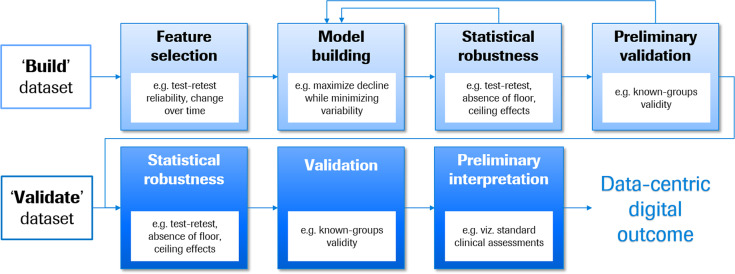
A roadmap to build and validate a data-centric digital outcome measure to monitor disease progression. A build dataset is used to select robust features for model building. The model building procedure is iterative, beginning with the generation of preliminary models tailored to the concept of interest, followed by tests for statistical robustness and preliminary validation. An independent validate dataset is used to confirm the statistical robustness of the model, validate that the model performs as expected, and support preliminary interpretation of the score magnitudes via comparisons with reference clinical assessments. Leftward arrows denote iterative processes.

The data-centric digital outcome measure procedure presented in Fig. 2 is designed to maximize sensitivity to detect disease progression, for example, of the core motor signs and symptoms associated with PD. The procedure assumes the availability of two independent sets of longitudinal sensor feature data from clinically and demographically matched patients, one to build and the second to validate the outcome measure, across a range of disease severities corresponding to the final intended use. The model-build process begins with a feature selection funneling process to ensure that the features provide information on progression that is above the level of measurement noise.

The selected features then enter a modeling process likewise tailored to the intended use of the outcome measure. First, the question of normalization should be addressed. The scales of the different sensor features should be examined to determine the relative meaning of point changes. Where reasonable, features should be normalized to the same scale prior to modeling, such that point changes have the same meaning across all sensor features. Minimally, scales should be adapted such that positive and negative values have the same clinical interpretation (e.g., better versus worse symptoms) to simplify the interpretation of the final model. Further, to minimize collinearity, the entire set of sensor features can be reduced to a smaller set of maximally informative sensor features using data reduction techniques. The tailored model building process should select multiple combinations and/or weightings of sensor features such that the final score represents maximal decline with minimal variability. These modeling approaches may include random search or genetic algorithms^26^. Cross-validation and boot-strapping assess the robustness of each candidate model and generate estimates of variability to facilitate model selection^27^, which can be based on two criteria: minimizing variability of the cross-validation (minimization criterion) and maximizing the change over time (maximization criterion), with equal weighting of these two.

We stress the importance of selecting a modeling process tailored to the intended use of the outcome measure, as opposed to optimizing the prediction of an existing standard clinical assessment. Models built using the former approach capitalize on the DHTT’s strengths, while models using the latter approach inherit the shortcomings of the existing clinical assessment and may neglect sensitive and novel features provided by the DHTT. For example, Zhan et al.^5^ developed the Mobile Parkinson Disease Score (mPDS) using an algorithm which weighted active test sensor features based on their response to dopaminergic medication. Thus, sensor features were selected in a data-driven approach independent of established clinical outcome measures such as the Movement Disorder Society—Unified Parkinson’s Disease Rating Scale^10^ (MDS-UPDRS) part III rating scale which sums physicians’ 0-to-5-point ratings of a number of motor symptoms. In the seven patients with pre- and post-medication DHTT and MDS-UPDRS assessments, the mPDS decreased by a mean of 16.3 (standard deviation 5.6) points (effect size = 2.9), while the MDS-UPDRS part III scores decreased by 10.4 (SD 4.6) points (effect size = 2.2) following dopaminergic therapy. While these findings are preliminary, we attribute the higher effect size of change in the mPDS compared to the MDS-UPDRS part III to the algorithm’s possibility to take advantage of the full informational content of the continuous sensor feature data, as opposed to the approach of using sensor feature data to predict changes in categorical MDS-UPRDRS part III scores.

The final steps in the model-build process ensures that the selected model has adequate statistical robustness (e.g., test–retest reliability, absence of floor and ceiling effects) and appears valid (e.g., “known-groups validity” whereby the measure distinguishes between patients of different severity levels). If inadequate, then an alternative candidate model from the model building procedure can be tested.

Model validity is confirmed and quantified in an independent group of demographically and clinically matched patients. The validation process entails the following steps:

1. Confirmation of statistical robustness: for disease monitoring measures, for example, test–retest reliability and the absence of floor and ceiling effects.
2. Validation: for example, known-groups validity.
3. Preliminary interpretation: an initial interpretation of model score magnitudes can be achieved by correlating these to standard clinical outcome measures. This is particularly important because DHTTs provide highly sensitive phenotypic data; therefore, the quantification of the magnitude of change corresponding to a generally accepted clinically relevant change on an existing COA facilitates interpretation of the DHTT score.

Thus, the data-centric digital outcome measure roadmap selects and integrates optimally appropriate and statistically robust sensor features into a model tailored to measure disease progression, and confirms the statistical robustness and validity in an independent sample.

### A roadmap to develop a patient-centric digital outcome measure

**Fig. 3:**
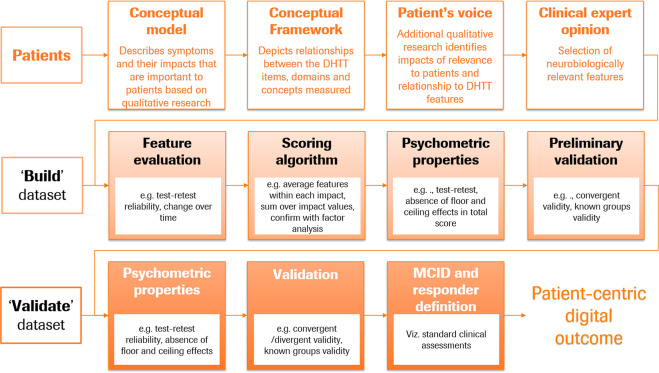
A roadmap to develop a patient-centric sensor-based outcome measure for an existing DHTT. Patient input provides the foundation for the development of this outcome measure. A conceptual model describes symptoms and impacts that are important to patients. A conceptual framework depicts the relationships between the concepts measured via the DHTT and associated items and domains. Additional qualitative research seeks to establish the impacts relevant to patients and their relationships with the DHTT features. Clinical expert opinions are required to ensure that the features represented in the final conceptual framework are neurobiologically relevant. The ensuing stages of outcome development require two independent datasets: the build dataset is used to select statistically robust and appropriate features, build a scoring algorithm, confirm psychometric properties of the scoring algorithm, and provide a preliminary validation; the independent validate dataset confirms the robustness of the psychometric properties, provides further validation data related to the DHTT outcome measure, and generates minimally clinically important difference (MCID) estimates and responder definitions.

The patient-centric digital outcome measure procedure described below aims to build and validate an outcome measure maximally sensitive to the motor symptoms directly relevant to patients’ everyday functioning, for use in measuring treatment effect in interventional studies or disease progression in longitudinal observational research studies (cf. Fig. 1). The patient-centric roadmap (see Fig. 3) builds upon the frameworks recommended by health authorities^15,17^. These guidelines are an invaluable resource to clinical researchers developing robust and fit-for-purpose measures for clinical research studies. The patient’s voice is central to this process^28^ and involves the generation of evidence via qualitative research studies which link the patients’ symptoms and impact experiences with specific DHTT sensor features. For example, in the context of PD, tremor is a symptom that is frequently reported as bothersome, particularly when patients complete activities of daily living (ADLs) such as dressing or holding a glass^28,29^, making tremor frequency an important symptom to measure.

A conceptual model is a visual representation of the symptoms patients deem important (e.g., hand tremor), and their impacts in everyday life (e.g., difficulty holding a knife and fork), and forms the foundation of the patient-centric approach. The conceptual model builds upon a number of different sources, e.g., literature reviews and qualitative research with patients, caregivers and treating physicians, and potentially social media analyses^17^. A conceptual model can be used to identify measures that adequately assess the concepts included in this model. In some cases, a measure may not exist or there may be an opportunity to improve upon existing outcomes and create something new. A conceptual model provides an important starting point to define the concept of interest (e.g., this could be focused on motor or non-motor symptoms or physical, social, or emotional impacts). Once the concept of interest has been defined, a conceptual framework can be created which depicts the relationship between the DHTT items, domains, and concepts measured. Its development is an iterative process, which relies on patient and where appropriate caregiver and physician input to converge on a final conceptual framework that adequately reflects the concept of interest under investigation and ultimately links the DHTT items, domains, and concepts with the final scores.

**Fig. 4:**
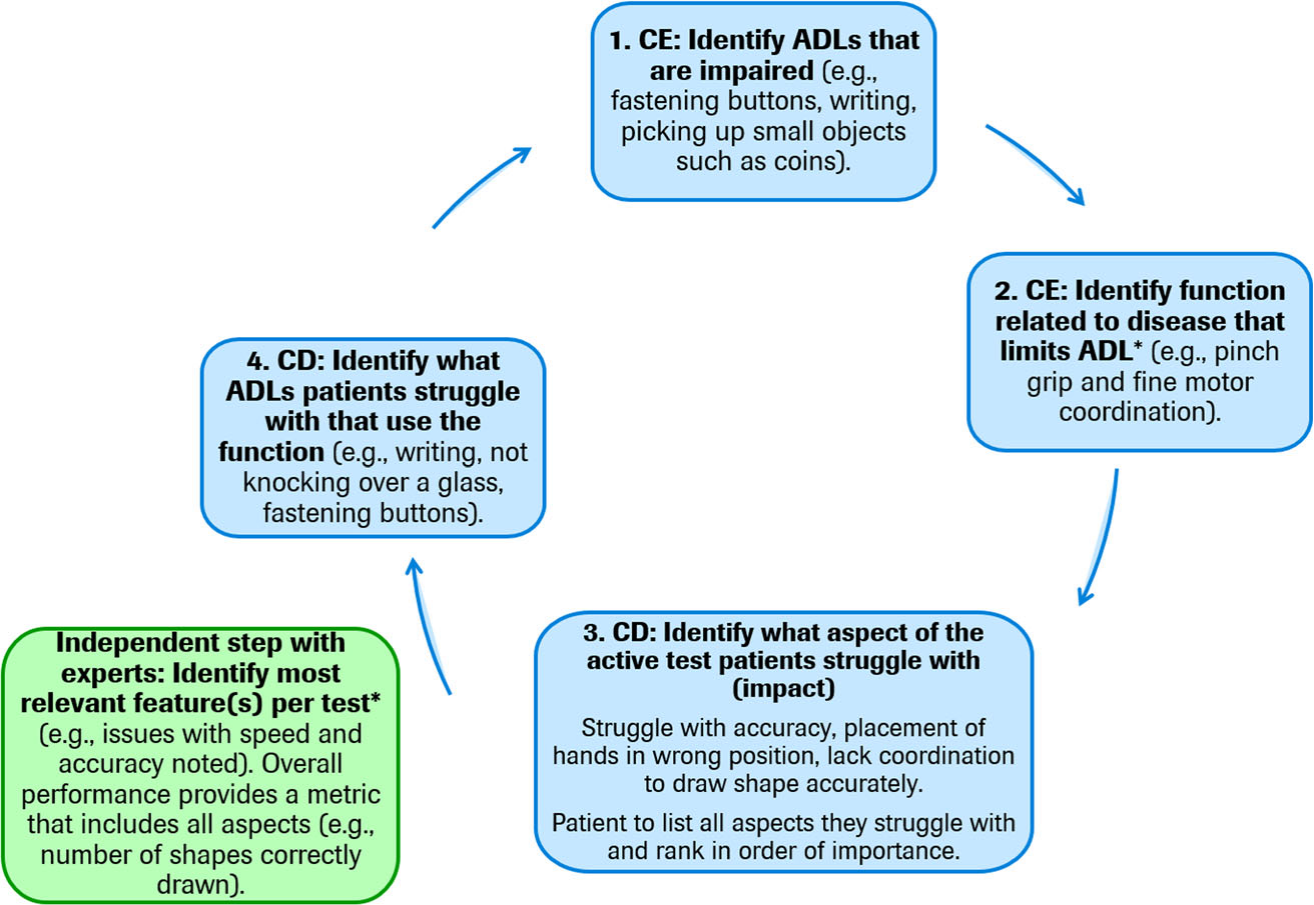
Path towards identifying the sensor features of an existing DHTT which correspond best to patient relevant symptoms and impacts. Traditional semi-structured interviews (i.e., concept elicitation (CE) involving the identification of activities of daily living (ADLs) that patients feel are impacted by their disease), followed by the identification of the disease symptoms related to each impaired ADL. In a third step, patients interact with the DHTT and complete a given task (e.g., draw a shape) and are asked to identify which aspects of the DHTT task present challenges, and finally, the relationships between the symptoms and challenges presented by the DHTT task are identified. The last two steps may include a cognitive debriefing (CD) interview to determine the patients’ understanding of the task instructions and further establish the relevance of the DHTT assessment. All other potential ADLs related to patients’ functions and DHTT sensor features should be discussed, represented by a return to the beginning of the interview procedure. The output of these interviews may lead to a refinement of the conceptual framework and associated DHTT if necessary, which should then be reviewed by experts (denoted by asterisk) to ensure a robust neurobiological mapping between symptoms and impacts and associated sensor features.

The development of a patient-centric digital outcome measure arguably requires novel qualitative concept elicitation and cognitive debriefing studies with patients, caregivers, and expert physicians to inform the creation of new measures based on appropriate patient-centric sensor features. However, in situations where a DHTT has been developed based on clinical insight and patient experience is derived subsequently, the most appropriate DHTT sensor features which correspond to patients’ symptoms and impacts can be identified during qualitative interviews and the adequacy of the conceptual framework revisited. Figure 4 illustrates one potential structure for concept elicitation interviews based on a developed DHTT using traditional semi-structured interviews^30^. In a first step, the interviewer identifies the ADLs that are impaired from a patient’s point of view. For example, an individual with PD may report problems with writing. Second, the disease symptom related to each limited ADL is identified. In the aforementioned example, the patient may report difficulties with coordination during writing. Third, patients interact with the DHTT and, in discussion with the interviewer, links between symptoms and the DHTT assessment are identified. For example, the individual with PD may recognize that he/she experiences similar motor difficulties while performing a DHTT assessment comprised of tracing figures on a smartphone screen as with writing. This step may also include a cognitive debriefing interview to determine the patients’ understanding of the task instructions and establish patients’ potential difficulties with the DHTT assessment. Fourth, the sensor feature(s) which best measure the patients’ difficulties is identified. In the present example, the interviewer may discuss with the patient whether sensor features measuring speed, accuracy, or a combination of both best reflects the difficulties experienced by the patient in everyday life. In a final fifth step, all other potential ADLs related to the function and sensor feature should be discussed in order for the discussion to return to ADL limitations. For example, does the patient experience similar difficulties with using cutlery, turning a key in the lock, using a keyboard, etc. This interview procedure may also be conducted with caregivers, in cases where patients do not accurately perceive or self-report a symptom or impact, but where these appear abnormal or worsening to caregivers. For example, patients may acquire an alternate frame of reference for judging their functioning, which may underestimate the magnitude of the symptom’s effect on daily life (e.g., patient may report that gait is not that disturbed [compared with several months ago, or given their coping ability], while the informant notes that patient’s gait activity is reduced by 50%).

The semi-structured interview data may be analyzed using thematic analysis methods^32^, whereby a coding process highlights and assigns participant quotes pertaining to specific symptoms and impacts on functioning to corresponding concept codes. Themes may be identified in the data both by topics emerging directly from the data (inductive inference) and by applying prior knowledge (abductive inference)^33^.

Finally, the conceptual framework should be reviewed by expert physicians and/or scientists to ensure that the mapping of symptoms and impacts to sensor features is clinically robust. The intended result of this qualitative study and expert interactions is a neurobiologically grounded conceptual framework which maps each disease symptom or impact to one or a number DHTT sensor features which measure the most relevant and meaningful impacts of the disease from the patient’s perspective.

Now that the content of the patient-centric digital outcome measure has been identified, the measure and associated relevant sensor features can undergo psychometric validation using two independent datasets, one to build and the second to validate the outcome measure (cf Fig. 3). Patients in the build and validate datasets should be comparable with respect to demographics and clinical characteristics, and cover the breadth of disease characteristics for which the outcome measure is intended.

Using the build dataset, features are evaluated for acceptable test–retest reliability and appropriateness for the intended use (i.e., in the case of measuring meaningful disease progression and detecting change over time). After sensor features that are not reliable have been removed, the outcome measure can be built with the aid of factor analytic methods. Factor analyses should be employed to understand the underlying structure of the measure (i.e., whether one or multiple domains exist) and ensure that the features assigned to each symptom/impact category indeed reflect the underlying concept. Moreover, factor analyses should ensure that the underlying factor structure indeed reflects the conceptual framework. Once redundancies have been minimized and the factor structure optimized, we propose to average all features within each symptom category, and to sum unweighted scores over symptom categories. In this way, no investigator bias is introduced into the score-building process. However, the scoring process should ultimately be driven by data reduction techniques such as factor analysis to understand whether a single sum score or multiple scores are supported.

The robustness of the patient-centric outcome measure and associated score should be assessed in the build dataset in a manner similar to that described for the data-centric digital outcome measure: its psychometric properties (e.g., test–retest reliability, absence of floor and ceiling effects), and preliminary validity (e.g., convergent validity, known-groups validity).

The final validation of the patient-centric digital outcome measure should occur with an independent sample of patients (“validate” dataset). The recommended procedure here mimics that for the data-centric digital outcome measure. Thus, the patient-centric digital outcome measure uses information from patients to select and integrate optimally appropriate and statistically robust sensor features into a model tailored to the patient’s experience, and confirms the statistical robustness and validity in an independent sample.

## Conclusions

There is to date no clear pathway for the development of robust and meaningful outcome measures based on DHTT sensor data. Here, we describe roadmaps to develop DHTT measures based on sensor data: the data-centric outcome measure is designed to quantify change in disease progression, while the patient-centric outcome measure is designed to quantify patients’ symptom experience. Both are designed for use in assessing disease progression and measuring treatment response in observational and interventional studies. As such, they may be applied to any DHTT data for use in any corresponding disease area. For example, the roadmaps could be applied in the well-established field of actigraphy to generate digital outcome measures to quantify sleep quality^21^ and symptom severity in mood-disordered patients^34^. Similarly, photoplethysmography data from wearables such as the Apple Watch could be explored with a data- and patient-centric approach to generate digital endpoints reflecting cardiovascular health^22^.

These roadmaps could also be amended for the development of, e.g., data-centric and patient-centric case-finding outcome measures. In this case, the differentiation of cases from controls would figure centrally in the feature selection process, model building and candidate model selection, and validation steps of data-centric and patient-centric digital outcome measures. Again, the roadmaps could be applied to generate case-finding outcome measures from any DHTT in any disease area. For example, such approaches are underway with smartwatch photoplethysmography data to identify individuals with potentially undiagnosed atrial fibrillation^35^, e.g., in the Apple Watch Study^36^, and to identify perceived stress levels^37^.

A critical issue in DHTT research is that of missing DHTT data, as they may result from disease-relevant factors which would then bias the final outcome measure. Examples are participants feeling particularly poorly or particularly well on a given day (and therefore unwilling to spend time interacting with the DHTT), thereby reducing the reliability and validity of the final digital outcome measure. Researchers are therefore encouraged to determine the reason for each set of missing data, ideally by directly asking participants when the patient logs onto the device following the missed assessment^17^. In any case, information should be obtained to estimate the proportion of missing data due to disease-relevant versus disease-irrelevant factors (e.g., technical problem with the device, family appointment interfered with testing), and thus the effect of disease-relevant missing data on the final outcome measure. Ideally, a second DHTT with maximal adherence (e.g., long-term body-worn sensors) would be additionally deployed to estimate the severity of symptoms on days where the regular technology was not used.

Several challenges remain in the optimized use of DHTTs in clinical research^6,7,11,14,31^. Normative data are required to understand the potential influence of demographic and general medical factors on DHTT sensor data, and to quantify the magnitude of impairment in patient populations. Moreover, standards should be developed for coding, sensor and data formats, and data analyses. The resolution of these issues will be greatly facilitated by the sharing of both raw and DHTT sensor feature data, whereby DHTT consortia (e.g. Critical Path for Parkinson’s (https://c-path.org/programs/cpp/), IDEA-FAST (https://idea-fast.eu/), MOBILISE-D (https://www.imi.europa.eu/projects-results/project-factsheets/mobilise-d), RADAR-AD (https://www.imi.europa.eu/projects-results/project-factsheets/radar-ad), Trials@Home (https://www.imi.europa.eu/projects-results/project-factsheets/trialshome) play an important role. Finally, it is clear that no single outcome alone is sufficient to monitor change in disease progression or to evaluate a treatment effect. It is our perspective that the combination of subjective (e.g., patient reported) and objective (e.g., DHTT wearable outcomes) data provides the richest source of information and insight into how a patient is feeling and functioning. Arguably, if an objective measure indicates change but the corresponding patient report suggests this change is not bothersome or detrimental to daily life, this provides important contextual information.

The field relies on regulatory authorities for guidance on the use of DHTTs in clinical drug development and for treatment decisions. The FDA, EMA and National Institute for Health and Care Excellence have issued guidances^38–42^ which acknowledge the fast-moving nature of this field. We believe that enough data and experience exist in the DHTT community to converge on a consensus on digital outcome measure development that respects the principles of established methods for outcome development^43^.

The present roadmaps are the result of more than five years’ work to develop and use DHTTs to evaluate the symptom experience in PD. It is hoped that the concepts outlined here will add to the scientific discourse towards an eventual consensus on the creation of novel digital outcome measures for both basic clinical research and clinical drug development. We invite all DHTT researchers to contribute digital data freely, and their insights as members of DHTT consortia to move this field forward together.
